# Synchronization of light flash with the irradiation pulse in proton beam therapy: A case report

**DOI:** 10.1016/j.tipsro.2023.100218

**Published:** 2023-07-07

**Authors:** Takashi Saito, Masashi Mizumoto, Yoshiko Oshiro, Toshio Miyamoto, Satoshi Kamizawa, Masatoshi Nakamura, Toshiki Ishida, Hirokazu Makishima, Haruko Numajiri, Kei Nakai, Takeji Sakae, Hideyuki Sakurai

**Affiliations:** a Department of Radiation Oncology, University of Tsukuba, Tsukuba, Japan; b Department of Radiation Oncology, Tsukuba Medical Center Hospital, Tsukuba, Japan

**Keywords:** Light flash, Proton beam therapy, Irradiation pulse, Quantitative

## Abstract

The correlation between sensory light flash and proton beam delivery was evaluated by measuring the timing of pulse beam delivery and light flash sensing using an event recorder in an 83-year-old patient receiving proton beam therapy (PBT) for nasopharyngeal adenoid cystic carcinoma. The treatment dose was 65 Gy (RBE) in 26 fractions with 2 ports, and both beams included the visual pathway (retina, optic nerve, chiasma). Measurements were obtained in 13 of the 26 fractions. The patient sensed a light flash in all 13 fractions and pressed the recorder button for 426 of the 430 pulsed beam deliveries, giving a sensing rate of 99.1%. The median duration of button-pressing of 0.3 s was almost the same as that of the beam pulse of 0.2 s, with a reaction time lag of 0.35 s. These results suggest a consistency between light flash during PBT and the timing of irradiation.

## Research highlight

• The proton beam pulse and the light sensing almost occurred simultaneously.
• The time between the proton beam pulse and the light sensing was comparable to the general visual reaction time.
• In the latter part of the treatment, the time between proton beam pulse and the light sensing tended to be shorter.

## Introduction

Light flash during radiotherapy is experienced by some patients and may be due to Cherenkov light or stimulation of the retina. Cherenkov light is produced when a charged particle travels through a transparent medium at a greater speed than light [1]. Tendler et al. demonstrated projected Cherenkov light in patients’ eyes during radiotherapy [2]. However, most previous studies have been based on self-reports of patients, and synchronization between the timing of proton beam delivery and light flash sensing is unclear. Therefore, we investigated the correlation of timing of beam delivery and light flash sensing objectively and quantitatively using a recording device in a patient who received proton beam therapy (PBT) for nasopharyngeal adenoid cystic carcinoma.

## Case presentation

### Patient and disease course

**Fig. 1:**
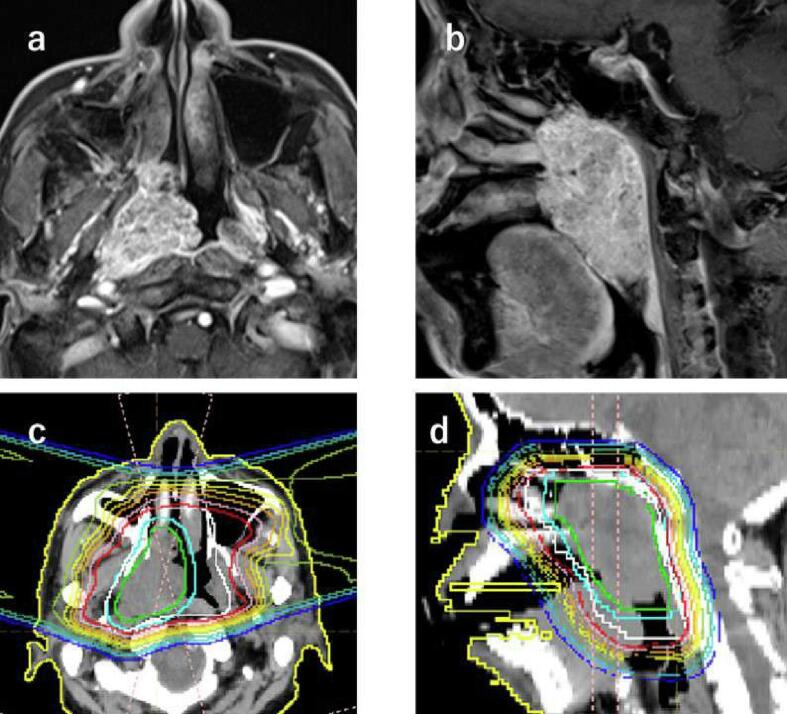
Contrast-enhanced T1-weighted MRI of a nasopharyngeal tumor and treatment planning for PBT. (a) Axial section of contrast-enhanced T1-weighted MRI. (b) Sagittal section of contrast-enhanced T1-weighted MRI. (c) Axial section for treatment planning. (d) Sagittal section for treatment planning.

An 83-year-old man visited our center with a chief complaint of epistaxis. Nasopharyngeal endoscopy revealed a mass in the nasopharynx, and a diagnosis of nasopharyngeal hemangioma was made based on gross findings and magnetic resonance imaging (MRI). The patient was followed up with periodic visits, but after 8 years, the tumor had enlarged and biopsy led to a diagnosis of adenoid cystic carcinoma. The tumor had partially invaded the skull base near the orbit (Fig. 1a, 1b). The patient opted for PBT after a head and neck surgeon determined that surgery would be difficult because the primary lesion was located in the nasopharynx. The patient had no medical history other than sinusitis, and the Karnofsky Performance Status was determined to be 90.

The treatment field included the entire nasopharynx. The clinical target volume (CTV) was set to include the tumor plus 5 mm. Passive-scattering PBT was delivered to the target with 2 ports (Fig. 1c, 1d). The retina and optic nerve were partially included in the irradiated area. Proton beam irradiation of 2.5 Gy (relative biological effectiveness, RBE) was administered 5 times a week for a total of 65 Gy (RBE) in 26 fractions. The energy of the proton beams was 200 MeV, which were delivered by the synchrotron every 2 s as a pulse of width 0.3 s. The dose rate was 1.9–3.1 Gy/min. The patient complained of light flash during the first session of PBT and sensed light flash in every treatment for two weeks, so we obtained his consent to participation in the current study. No sound or light was generated in the irradiation chamber during the PBT sessions.

### Recording of light flash sensing

**Fig. 2:**
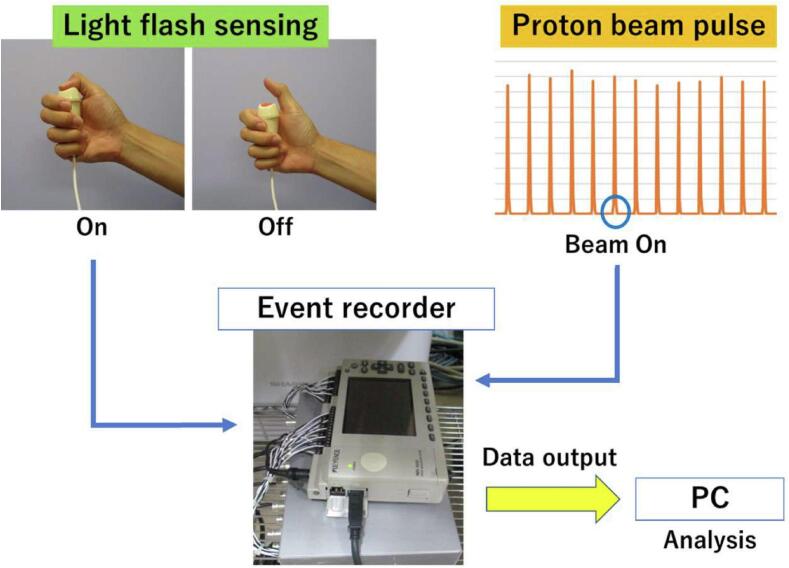
Recording method and flow of information to the event recorder.

**Fig. 3:**
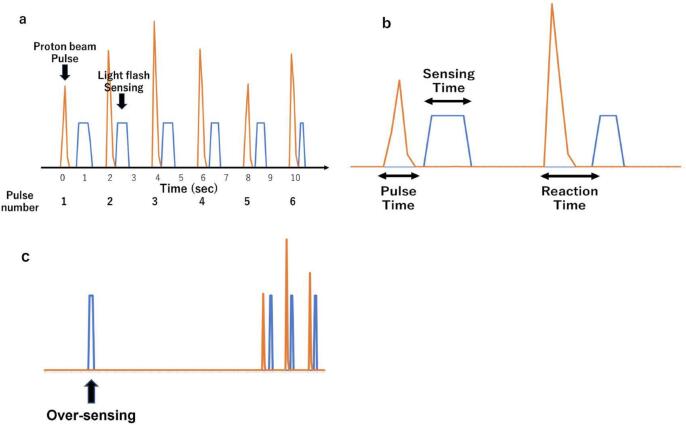
Details and definitions used in recording. (a) Time recording of proton pulse and sensing light flashes. (b) Definitions of pulse time, sensing time and reaction time. (c) Definition of over-sensing.

We made a small recorder based on a modified nurse call and asked the patient to push the recorder button when he sensed a light flash, and to release the button when the light flash disappeared. As the patient pushed the button of the recorder, an electrical signal of light flash sensing was sent to the event recorder. Similarly, the proton beam pulse was transferred as an electrical signal to the event recorder, so that each signal was recorded as a separate event (Fig. 2). The recorded events were sent to a PC with time information and analyzed on the PC. These measurements were achieved in 13 of the 26 fractions. The pulse time was defined as the time of proton beam irradiation, the sensing time as the time the patient was pressing the button, and the reaction time as the time between the start of proton beam irradiation and the time the patient started to press the button (Fig. 3a-c).

## Results

**Fig. 4:**
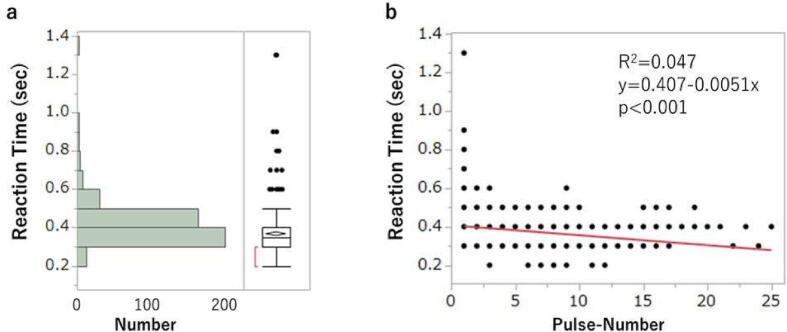
Histogram of reaction time and correlation between pulse number and reaction time. (a) Histogram of reaction time in all sessions. (b) Correlation between pulse number and reaction time in Spearman correlation analysis.

Of the 430 pulsed proton beams, 426 sensations of light flash were confirmed, giving a sensing rate of 99.1%. The rate at which the button was pressed by the patient without proton beam irradiation (over-sensing rate) was only 0.43%. The median pulse time, sensing time and reaction time were 0.3 s (range: 0.1–0.4 s), 0.2 s (0.1–0.6 s) and 0.35 s (0.2–1.3 s), respectively. The histogram of reaction time for all sessions and the correlation between pulse number and reaction time in Spearman correlation analysis are shown in Fig. 4. A reaction time of 0.3 to 0.4 s occurred for 364 cases (86%) and there was a tendency for reaction time to decrease from the beginning to the latter half of each session.

## Discussion

Light flashes have been described in irradiation of head and neck tumors, but evidence for this phenomenon is often based on personal statements of patients [3], [4], [5], [6], [7], [8]. Wilhelm-Buchstab et al. recorded light flash sensing using an event recorder and analyzed the relationship between visual sensation and the treatment beam path [9], with the finding that light flash had the highest probability when the optic chiasm and optic tract were included in the beam path. However, as far as we are aware, a relationship between the timing of a light flash sensation and beam on/off has not been suggested previously. Using a proton beam delivered in a pulsed fashion, we were able to examine the relationship between pulse timing and the duration of light sensing objectively, even though no light or sound is generated within the treatment room. Our results suggested that light flashes were correlated with the pulse of proton beam irradiation.

The median reaction time was 0.35 s, which is the time lag between the start of the proton beam pulse and the patient starting to press the button. Generally, visual reaction time is 0.2–0.4 s and tends to be slower in elderly people [10]. The patient was 83 years old, so this time lag is consistent with the general visual reaction time in elderly people. Repetition of similar stimuli is known to shorten the reaction time [11] and this tendency was also observed in this case. The fact that the reaction time was within the general visual reaction time range and was shortened by repeating the same stimuli supports the idea that proton beam irradiation is perceived through the visual pathway.

Cherenkov light is generally produced less frequently during PBT because the energy of proton beams is too low. The energy required to produce Cherenkov light by the proton beam itself is estimated to be > 482 MeV [12] and the energy of the proton beam is 155–250 MeV at our center, which is far below this threshold. However, some reports have suggested sensing of light flash during PBT for the head or head and neck region [3], [4]. Helo et al. suggested that direct stimulation of the retina by treatment beams or secondary electrons may influence light flash [13]. The minimum energy of a proton beam required to generate Cherenkov light via secondary electrons is 120 MeV, which can be achieved with the proton beam at our center. In this case, all beams included visual pathways such as the retina, optic nerve, and chiasma; therefore, it becomes challenging when we attempt to determine if the light flash is caused by direct stimulation or by the influence of secondary electrons. However, the time of generation of secondary electrons is several nanoseconds in PBT [13], and thus, the time lag with beam delivery is almost negligible. Given that the time lag between beam delivery and the light flash sensation in this case matches the general visual reaction time, it suggests that the idea of secondary electrons inducing light flash sensation does not conflict with our findings. Nevertheless, more comprehensive research is required to fully unravel the process underlying this observed phenomenon.

Definitive conclusions cannot be drawn from a single case report, but our finding that light flash coincided with the proton beam pulse is of interest. Use of the same method for more patients receiving PBT with various ports will allow a more detailed analysis of light flash, including the dose threshold and related irradiation sites.

## Conclusion

The results of this study show that light flashes during proton beam therapy and the timing of irradiation pulses are synchronized.

**Declarations**

Ethics approval and consent to participate: Informed consent was obtained from the patient.

Consent for publication: The patient consented to publication of this report.

Availability of data and materials: The datasets used and/or analyzed during the current study are available from the corresponding author on reasonable request.

Authors' contributions: All authors read and approved the final manuscript.

Funding: This work was supported by the University of Tsukuba.

## Declaration of Competing Interest

The authors declare that they have no known competing financial interests or personal relationships that could have appeared to influence the work reported in this paper.
